# Clinical relevance of the tumor microenvironment and immune escape of oral squamous cell carcinoma

**DOI:** 10.1186/s12967-016-0828-6

**Published:** 2016-04-05

**Authors:** Alexander W. Eckert, Claudia Wickenhauser, Paul C. Salins, Matthias Kappler, Juergen Bukur, Barbara Seliger

**Affiliations:** Department of Oral and Maxillofacial Plastic Surgery, Martin-Luther-University Halle-Wittenberg, Ernst-Grube-Str. 40, 06120 Halle (Saale), Germany; Institute of Pathology, Martin-Luther-University Halle-Wittenberg, Magdeburger Str. 8, 06110 Halle (Saale), Germany; Mazumdar Shaw Cancer Center and Narayana Hrudayalaya Multi Specialty Hospital, 258/A, Bommasandra Industrial Area, Bangalore, 560099 India; Institute of Medical Immunology, Martin-Luther-University Halle-Wittenberg, Magdeburger Str. 2, 06110 Halle (Saale), Germany

**Keywords:** Oral squamous cell carcinoma, Hypoxia, Metabolic adaption, Immune escape, Immune response

## Abstract

**Background:**

Changes in the tumor microenvironment and immune surveillance represent crucial hallmarks of various kinds of cancer, including oral squamous cell carcinoma (OSCC), and a close crosstalk of hypoxia regulating genes, an activation of chemokines and immune cells has been described.

**Methods:**

A review about the pivotal role of HIF-1, its crosstalk to various cornerstones in OSCC tumorigenesis is presented.

**Results:**

Hypoxia is a frequent event in OSCC and leads to a reprogramming of the cellular metabolism in order to prevent cell death. Hypoxic OSCC cells induce different adaptive changes such as anaerobic glycolysis, pH stabilisation and alterations of the gene and protein expression profile. This complex metabolic program is orchestrated by the hypoxia inducible factor (HIF)-1, the master regulator of early tumor progression. Hypoxia-dependent and -independent alterations in immune surveillance lead to different immune evasion strategies, which are partially mediated by alterations of the tumor cells, changes in the frequency, activity and repertoire of immune cell infiltrates and of soluble and environmental factors of the tumor micromilieu with consecutive generation of an immune escape phenotype, progression of disease and poor clinical outcome of OSCC patients.

**Conclusions:**

This review focusses on the importance of HIF-1 in the adaption and reprogramming of the metabolic system to reduced oxygen values as well as on the role of the tumor microenvironment for evasion of OSCC from immune recognition and destruction.

## Characteristics of oral squamous cell carcinoma and additional prognostic markers

Oral squamous cell carcinoma (OSCC) are among the ten most frequent human malignancies and mostly arise in the context of alcohol abuse and smoking while HPV infection does not play an important role in carcinogenesis of this disease [1]. As for the oral cavity SCCs, many authors reported frequent high-risk HPV involvement by considering the over-expression of p16INK4A as equivalent to HPV infection [2, 3]. Nevertheless, recent data in oral cancers indicate that p16INK4A over-expression is due to different mechanisms and high-risk HPV infection is very rarely detectable in OSCCs [2, 4, 5].

Despite some progress in diagnostics and therapeutic options the 5-years overall survival rate (OSR) stagnates up to only 40–50 % over a 40-years period [6–9].

However, the routinely used tumor stratification based on the tumor, node, metastasis (TNM) classification together with the histological grading alone is not sufficient to predict the individual prognosis of an OSCC [10, 11]. Therefore, there is an urgent need to establish additional prognostic factors. Interestingly, the aggressiveness of OSCC increased with aboral localization. Furthermore, systematic analysis of the expression profiles of OSCC led to the identification of different tumor markers with prognostic value in OSCC, such as the carcinoembryonic antigen (CEA), carbonic anhydrase (CA) 19–9, CA 125, CA 15–3 and the squamous cell carcinoma (SCC) antigen [12]. Recently, also hypoxia-associated genes were identified as important markers for prognosis of OSCC [10, 13] suggesting that hypoxia-dependent pathways including the stabilization of HIF-1α play a key role in the development and progression of this disease [14–16]. So far, the expression and localization of HIF-1α, the key regulator of hypoxic responses, in OSCC cells have been determined by several groups, although the downstream effects in particular the expression of hypoxic pathways in the context of OSCC progression are widely unknown. In addition, the different cellular processes controlling the composition of immune cell infiltration and immune surveillance have to be elucidated to develop novel prognostic markers and potential therapeutic strategies. Recently, the molecular mechanisms and signal transduction pathways involved in the development of head and neck squamous cell carcinoma (HNSCC) in general and of OSCC in particular have extensively reviewed [17, 18]. Therefore, this survey summarizes the molecular biologic and immunologic aspects involved in OSCC with a specific focus on the hypoxic tumor microenvironment and its link to an altered tumor metabolism.

## Tumor hypoxia and prognosis in OSCC

### HIF-1—a key regulator protein in cancer progression

All mammalian cells require oxygen for their essential metabolic program including oxidative phosphorylation [19, 20]. The normal physiological oxygenation of mammalian tissues varies between 1 and 11 % (approximately partial pressures of 7.5–85 mm Hg) [21, 22]. Intra-tumoral hypoxic stress could be mediated by rapid cell division, aberrant angiogenesis and blood flow. An oxygen pressure below 5–10 mm Hg is a powerful force for metabolic adaption and leads to structural alterations favouring survival, angiogenesis and progression of tumors, epithelial-mesenchymal transition (EMT), suppression of immune reactivity, which then correlated with poor prognosis and therapy resistance of tumor patients [23–25]. Hypoxic stress induces a complex gene expression program [26, 27] with HIF-1 as a master transcriptional regulator of genes controlling oxygen homeostasis [28], thereby mediating cellular and systemic adaptive responses to maintain oxygen homeostasis in all metazoan species [29].

The HIF system consists of three principal molecule groups: The most important molecule is HIF-1, which contains the oxygen sensing subunit alpha (HIF-1α). Next to HIF-1α HIF-2α and various homologues of HIF-3α have been identified [24, 28, 30, 31]. Despite HIF-2α is also regulated by an oxygen-dependent hydroxylation [24], it is not involved in all types of cancer [32, 33]. In some carcinoma, an inverse expression of HIF-1α and HIF-2α was found. While HIF-1α is overexpressed and associated with disease progression in HIF-2α expression is low and is a good prognostic marker [34]. In contrast, the third homologue, HIF-3α, may function as an inhibitor of both HIF-1α and HIF-2α [24].

HIF-1 is a basic helix-loop-helix-PAS heterodimer composed of an alpha and a beta subunit [19, 22]. The beta subunit, also called aryl hydrocarbon receptor nuclear translocator (ARNT), is a constitutively expressed 91–94 kDa protein [20, 30]. Under physiologic conditions HIF is hydroxylated by specific prolyl hydroxylases (PDH) on proline residues 402 (Pro-402) and 564 (Pro-564), which facilitates the binding of the von Hippel-Lindau tumor suppressor protein (pVHL) to HIF-1α/HIF-2α leading to a rapid ubiquitination by an E3-ubiquitin ligase complex followed by the proteasomal degradation of HIF1α/HIF2α [32]. Another possibility to inhibit HIF-1α is mediated by the factor inhibiting HIF-1 (FIH-1α), which hydroxylates asparagine 803 in the transactivation domain and consequently blocks the binding of the co-activators p300 and CBP [28]. Under hypoxic conditions, HIF-1α dimerizes with HIF-1β and the resulting transcription factor HIF1 activates a large panel of target genes. HIF-1α represents a central protein involved in different pathways that are important for the survival of cancer cells in early cancer disease progression. Recently, a meta-analysis of 28 studies was performed on a large cohort of overall head and neck cancer (HNC) patients demonstrating an association of HIF-1α and HIF-2α overexpression with mortality particularly in the Asian population wherein increased levels of HIF-1α were associated with a reduced survival [15]. This meta-analysis further clarified that HIF-1α expression has a distinct prognostic value in the different subtypes and localization of HNC [15] indicating that the different subtypes of HNC should be analysed separately. Concerning OSCC increased HIF-1α levels have been shown to correlate with poor prognosis [14, 35–37].

### HIF-1α target genes

**Fig. 1:**
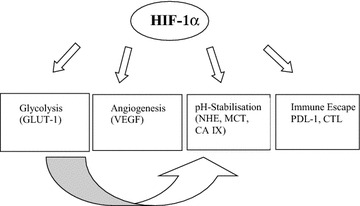
General pathways in oral squamous cell carcinoma as downstream products after hypoxic stabilisation of HIF. *GLUT-1* Glucose transport molecule -1, *VEGF* Vascular endothelial growth factor, *NHE* Natrium/hydrogen exchanger, *MCT* Monocarboxylate transporter, *CA IX* Carbonic anhydrase 9, *PDL-1* Programmed cell death ligand, *CTL* Cytotoxic T lymphocyte

A more general finding was first proposed by Semenza and co-workers demonstrating that HIF-1α controls the expression of 100 genes involved in tumor progression [38] and this number increased to more hundred genes during the last years [39]. These genes include the CAIX, a membranous enzyme involved in pH regulation [40] the glucose transporter-1 (GLUT-1) responsible for glucose import [41] and the monocarboxylate transporter-1 (MCT-1) and MCT-4 important for lactate transport [42, 43]. Furthermore, HIF-1 targets are not only involved in glucose transport and glycolysis, but also in cell survival and proliferation, invasion and metastasis formation [24]. This is in line with reports describing a hypoxia-mediated “angiogenic” or “glycolytic” switch of tumor cells [44]. Furthermore, a continuous low O_2_ partial pressure has an impact on both the cellular metabolism and HIF signaling [22]. The HIF-1-mediated pathways, which play an important role in stabilization and/or progression of OSCC lesions, are (i) glycolysis (ii) angiogenesis (iii) pH stabilisation (iv) the microenvironment and epithelial mesenchymal transition (EMT) and (v) distinct strategies of tumor cells to escape immune surveillance (Fig. 1).

#### Role of glycolysis

Hypoxia induces adaptive changes in the cellular metabolism by HIF-1 as a master regulator to balance oxygen supply and demand [34]. OSCC cells obtain most of their energy by glycolysis. Since glycolysis delivers only 2 ATP molecules compared to 38 ATP molecules by respiration, an increased glucose uptake is essential for tumor cells to survive [45]. The family of glucose transporter molecules summarizes 13 members [46, 47]. The most investigated transport molecules in OSCC are HIF1 and GLUT-1 [41, 48]. Own investigations demonstrated a significant correlation between increased glucose uptake and poor prognosis in OSCC [49]. Similar results were obtained by Harshani [48]. In addition, the hypoxia linked upregulation of GLUT-1 was also described by Gimm and co-authors, which negatively interfered with the survival of OSCC patients [50]. An increased glucose consumption leads to an acidification of tumor cells. The next crosstalk to enable tumor cell survival is an upregulation of carbonic anhydrase(s). This was accompanied by co-expression of HIF-1α and CAIX, from which the latter is also transcriptionally activated by the HIF complex. Interestingly, the risk of tumor-related death for the patients’ groups with the worst prognosis was comparable independent of HIF-1α alone (RR = 4.53) [51]. In addition, GLUT-1 is overexpressed at a high frequency in OSCC lesions, patients with tumour lesions expressing both HIF-1 α and GLUT-1 had a 5.13-fold increased risk of tumour-related death (P = 0.017). Co-expression of high levels of HIF-1α and GLUT-1 was hence significantly correlated with poor prognosis in OSCC patients. Since proteins associated with the glucose and lactate metabolism often co-localize in hypoxic areas of OSCC [52, 53], a combined analysis of the expression pattern of both proteins might be used as an early diagnostic and independent prognostic marker [54]. Moreover, enhanced glucose uptake by OSCC cells reduced the sensitivity of tumor cells to cisplatin-based chemotherapy [55].

#### Role of angiogenesis

Tumor progression is a multifactorial process including the induction of angiogenesis and cancer cell proliferation in OSCC cells. This was accompanied by an upregulation of diverse angiogenic markers. Angiogenin expression significantly correlates with HIF-1α [56] and with an increased microvessel density (MVD). When OSCC cells were cultured under mild hypoxia (5 % O_2_) only HIF-2 α contributed to VEGF-expression. In contrast, at 1 % O_2_ VEGF’s were regulated by both HIF-1 α and HIF-2α. As a consequence both HIF-1α and HIF-2α play a pivotal role in tumor angiogenesis and tumor growth of OSCC [37]. In addition, HIF-1α is involved in tumor lymphoangiogenesis. This was demonstrated by analysis of the density of blood and lymphatic microvessels in OSCC using immunohistochemical staining for CD43 and LYVE-1: HIF-1α overexpression significantly correlated with a VEGF-C upregulation. Consequently, a higher lymphatic vessel density was found in HIF-1α-positive OSCC [57].

#### Role of pH stabilisation

**Fig. 2:**
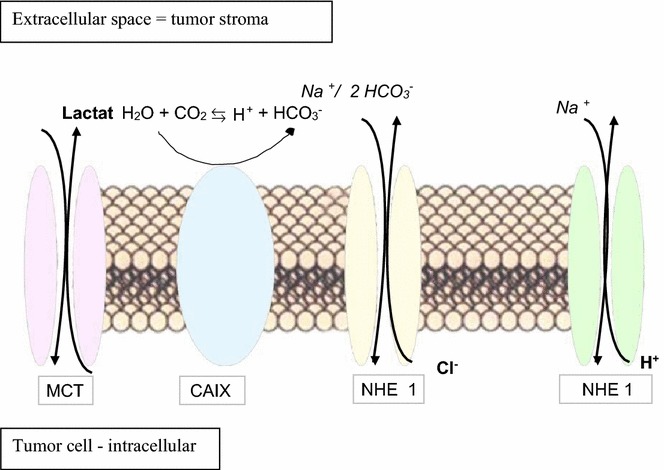
HIF-triggered complex system for pH stabilization. *MCT* Monocarboxylate exchanger, *CAIX* Carbonic anhydrase 9, *NHE* *1* Sodium/hydrogen exchanger

The proliferation of cancer cells creates toxic waste products and an acidification leading to a decrease in the intracellular pH of tumor cells. The metabolic adaption accumulates different ionic exchangers at the tumor cell membrane to maintain intracellular pH (pHe) (Fig. 2). Dysbalances in pHe have been shown to be associated with cancer progression [58]. Moreover, HIF-1α orchestrates also pH stability of the tumor cells [59] and extracellular matrix adaption [60, 61], which is linked to alterations of the metabolic program by affecting the expression of the HIF-regulated pathway components [59]. This includes e.g. an upregulation of CAIX, which is associated with nodal metastases and a decreased survival of OSCC patients [62]. The deregulated pH in OSCC is also an adaptive feature, which could be divided into general pathways. First it is necessary to maintain the intracellular pH (pHi). Second, an acidification of extracellular pH (pHe) is the consequence. In normal differentiated adult cells, intracellular pH (pHi) is generally ~7.2 and lower than the extracellular pH (pHe) of ~7.4. However, cancer cells have a higher pHi of ≥7.4 and a lower pHe of ~6.7–7.1. A complex membrane spanning system is initiated to emerge pH homeostasis. In this process HIF-1α is a sensitive controller and regulator of a complex adaptive metabolic response to alterations in particular the intracellular pH value [33, 62]. An increased pHi is permissive for cell proliferation and the evasion of apoptosis, facilitates metabolic adaptation and is obligatory for efficient directed cell migration [58]. It is proposed that the hypoxic microenvironment induced the epithelial to mesenchymal transition (EMT), upgraded stem-like properties, promoted invasion and metastasis, and thus increased the malignancy. Several pluripotency transcription factors seem to be associated with the EMT transition process like Nanog and POU5F1, SNAI and SOX-2 [11, 63–66].

In our opinion, the same effects are another hallmark of OSCC development as described by Guo and co-workers for gastric cancer [67, 68]. The HIF-1α-mediated hypoxia-dependent down-regulation of E-cadherin and upregulation of N-cadherin is caused by the activation of the transcription factor SNAI2 thereby promoting EMT of tumor cells. Therefore, the aberrant HIF-1α and SNAI2 expression combined with the cadherin switch has been suggested as potential risk marker for predicting metastasis and clinical prognosis [68]. Furthermore, the switch to anaerobic metabolism the genetic instability of OSCC cells greatly increases [44].

## Tumor microenvironment and epithelial-mesenchymal transition (EMT)

When hypoxia results in epithelial mesenchymal transition, the resultant reduction in adherence and the change of cytoskeleton enables migration leading to metastasis. The hostile microenvironment, which is created by hypoxic events, combined with the presence of growth factors and cytokines are the crucial initiating events leading to this complex transition. EMT is classified as three different subtypes based on different biological settings, which causes various functional consequences. Type 1 EMT is considered to be associated with implantation, embryogenesis, and organ development. Type 2 EMT occurs in organ fibrosis, whereas Type 3 EMT occurs in carcinogenesis [69]. During the progression to metastatic competence, the OSCC cells enter into a metabolic and survival reprogramming process. This allows the OSCC cells to acquire features similar to mesenchymal cells that may significantly impart invasiveness, changes in adhesive properties, activation of motility and the degradation of the extracellular matrix. Major signaling pathways, which are commonly implicated in epithelial-mesenchymal transition, include TGF-β, Wnt, Notch, Hedgehog, and others. These pathways converge on several transcription factors, including zinc finger proteins Snail and SNAI2, Twist, ZEB1/2, and Smads. These factors interact with one another and others to provide crosstalk between the relevant signaling pathways [70].

Although hypoxia-induced EMT signalling occurs in all tumor cell populations, only the stem-like cells acquire high migratory potential, which suggests that these cells are potentially responsible for invasion and consequently metastasis [71]. Furthermore, in the presence of hypoxia, the utilization of epigenetic mechanisms of gene regulation could play an important role in aggressive behaviour of tumors.

The tumor microenvironment appears to play a prominent role in affecting EMT changes. For example, the E-cadherin transcriptional repressor, *TWIST*, is positively regulated by HIF-1α [72]. It has been demonstrated that hypoxia-induced EMT in in vitro experiments is associated with increased HIF-1α and Twist expression that knock-down either HIF-1α or Twist blocks hypoxia-induced EMT [73].

There are many pathways regulating EMT. Amongst them are Notch signaling and Wnt pathways, which have been shown to be involved in the conversion of the hypoxic stimulus into EMT. They are also important in increasing both motility and invasiveness of the tumor cells [74]. The EMT program has been found to be active in the invasive front of OSCC. There is evidence of reduced E-cadherin expression at the invasive front of OSCC. Additionally, there is an association with histological invasiveness, which suggests that this protein could be a potential EMT marker, thus offering prognostic information in OSCC [75].

Moreover, there is further evidence that suggests hypoxia induced EMT in OSCC cell lines via activation through Notch signaling. Therefore, inhibition of the Notch signaling pathway to suppress EMT is possibly a useful approach for the treatment of OSCC [76]. An understanding in how hypoxia regulates EMT related transcriptome could help to identify nodes of interaction related to cancer progression. Consequently, further targeting hypoxia induced EMT could show to be an alternative therapeutic approach for the prevention and treatment of OSCC.

## Distinct strategies of tumor cells to escape immune surveillance

A novel and recently emerging aspect in tumorigenesis is the crosstalk between hypoxia/HIF-1α and immune recognition/immune escape mechanisms [77]. Hypoxia has been shown to influence the repertoire and activity of immune cells in various tumor entities, including OSCC. Different strategies by which hypoxia contributes to tumoral immune escape from recognition by NK cells and cytotoxic T lymphocytes have been described [78–80].

**Fig. 3:**
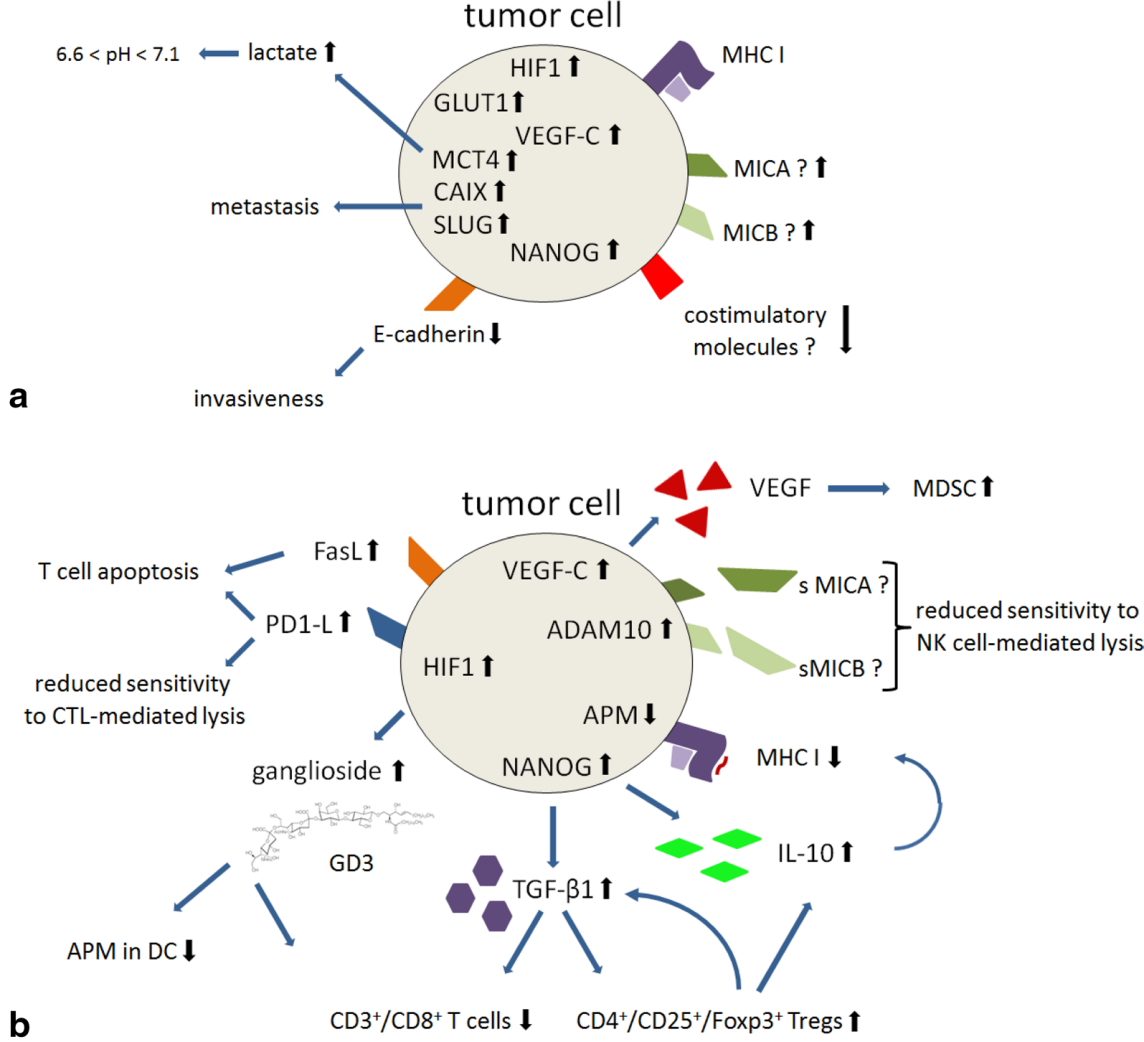
Complex system of adaption to hypoxic stress in early carcinogenesis (**a**) and mechanisms for immune escape (**b**)

**Fig. 4:**
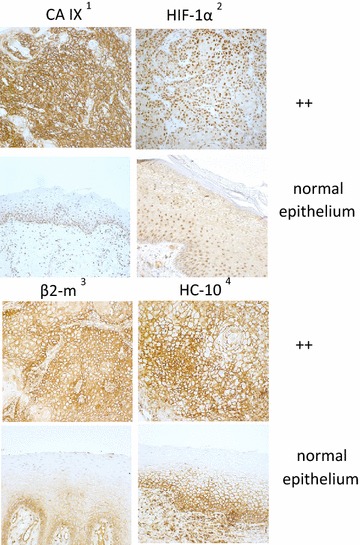
Differential expression of hypoxia markers and HLA class I in OSCC-carboanhydrase *(CA) IX* ^1^, hypoxia inducing factor *(HIF) 1α* ^2^, beta2-microglobulin *(β2-m)* ^3^ and heavy chain *(HC)-10* ^4^ in human head and neck squamous cell carcinoma (OSCC)

Hypoxic stress is known to induce a variety of immune suppressive molecules, such as IL-10 and TGF-β (Figs. 3, 4). This step could further induce the differentiation of tumor-associated macrophages (TAMs) into M2 macrophages to suppress anti-tumoral activities [78]. In addition, HIF-1α orchestrates the CD4^+^ and CD8^+^ T-cells regarding their survival, apoptosis and cytokine secretion. In HIF-knock down mice an increased frequency and activation of CD4^+^ and CD8^+^ T cells was found, which produced higher levels of IFN-γ thereby enhancing anti-tumor responses [81]. Furthermore, the interaction of HIF-1α with the cytotoxic T cell lymphocyte-antigen 4 (CTLA-4) as immune checkpoint regulator and its receptors CD80/CD86 is well known. The HIF-triggered CTLA-4 blockade caused a reduction in the frequency of tumor infiltrating Tregs [78]. Another key role of HIF-1α associated with immune escape and induction of tolerance is the shedding of cell surface immune checkpoint regulators like MICA, which interact with the NKG2D receptors of different immune cells. Tumor cytolysis is avoided due to the resistance to NK cell attack [82]. This immune adaption is dependent on the HIF-1α-mediated induction of metalloproteinase ADAM-10 in tumor cells [83].

In addition, PDL-1 is expressed under hypoxic conditions leading to resistance to CTL-mediated lysis. Moreover, the cellular co-localisation of HIF-1α and PDL-1 in tumors has been well investigated [79]. In advanced OSCC Chen and co-workers described positive staining for both HIF-1α and PDL-1, which was associated with a worse prognosis [84]. In addition, a binding of HIF-1α to the hypoxia-response element of the PDL-1 promoter has been shown in both breast and prostate cancer cell lines demonstrating a link of HIF-1α with the checkpoint inhibitor [79].

It is generally accepted that tumor cells could be recognized as abnormal cells and thus be destroyed by immune cells. However, tumors have developed different mechanisms that allow their immune escape such as deficient expression of tumor antigens on their cell surface, loss or a reduced expression of MHC (major histocompatibility complex) class I molecules, lack of expression of co-stimulatory molecules, production of immune suppressive molecules like the transforming growth factor (TGF)-*β*, IL-6, IL-10, prostaglandin (PG) E2 and adenosine, resistance to apoptosis, and/or the expression of Fas ligand (FasL), which leads to the death of tumor-infiltrating lymphocytes (TILs) as well as induction of co-inhibitory molecules of the B7 family and of non-classical HLA class I antigens, e.g. HLA-G and HLA-E [85–88]. In addition, tumor cells can recruit TAMs, the major inflammatory components of the tumor microenvironment by secreting the colony stimulating factor (CSF-1), the chemokine ligands 2, 3, 4, 5, and 8 (CCL2, 3, 4, 5, and 8) and VEGF [89–91]. Due to their distinct functions macrophages could be divided into (i) the M1 phenotype, which kill pathogens, promote the activation of cytotoxic CD8^+^ T cells and the differentiation of naïve CD4^+^ T cells into Th1 effector and Th17 cells and (ii) the M2 macrophages, which stimulate CD4^+^ Th2 cells as well as regulatory T cell differentiation and promote angiogenesis and tissue remodeling (pro-tumor functions). The effect of Th17 cells is controversially discussed. However, so far no information exists on the prognostic impact of premalignant oral lesions developing OSCC and their TAM phenotype. It might be an advantage to limit the progression of premalignant lesions to cancer by sustaining the Th17 phenotype [92]. This is in contrast to Deng and co-authors demonstrating a recruitment and proliferation of Th17 cells in the intestine promoting colon cancer [93]. Multiple studies demonstrated a correlation between the frequency of macrophages in the tumor microenvironment and the patients’ prognosis. This is due to the secretion of the epidermal growth factor (EGF), platelet-derived growth factor (PDGF), TGF-*β*, IL-6, IL-1, and tumor necrosis factor (TNF)-*α* of TAMs, which creates a favorable milieu for tumor growth. In hypoxic areas, TAMs stimulate angiogenesis by secreting TGF-*β*, VEGF, granulocyte macrophage (GM)-CSF, TNF-*α*, IL-1, IL-6, and IL-8, promote tumor cell migration and invasion via matrix metalloproteinases (MMPs), TNF-*α*, and IL-1 and induce immunosuppression via TGF-*β*, PGE2, and IL-10. T lymphocytes could also contribute either to tumor cell destruction or facilitate its development. While Th1 CD4^+^ T cells facilitate tumor rejection by assisting the function of cytotoxic CD8^+^ T cells, Th2 CD4^+^ T cells promote antibody production of B cells by secreting cytokines. The CD4^+^ T regulatory cells (Tregs) expressing FoxP3 promote tumor progression by inhibiting the NK and T cell functions. Their frequency is increased in tumor patients and associated with a worse prognosis. In addition, the number of myeloid-derived suppressor cells (MDSCs), which are induced by VEGF, GM-CSF, TGF-*β*, IL-6, PGE2, and cyclooxygenase (COX)-2 are often increased in tumor patients. MDSCs are a heterogeneous population of immature and progenitor myeloid cells with an immunosuppressive role in various types of cancer, including OSCC. A significant accumulation of both granulocytic and monocytic MDSC was observed in head and neck cancer. The frequency of granulocytic MDSC showed an inverse correlation to the frequency of T cells in the peripheral blood. The increased granulocytic MDSC significantly associated with advanced clinical stage and poor prognosis of head and neck cancer patients [94]. Cetuximab treatment of HNSCC significantly increased monocytic MDSC in non-responders, but decreased granulocytic MDSC in responders of HNSCC patients. The frequency of MDSC known to promote tumor progression correlate with poor prognosis in cancer patients including HNSCC [95].

They have been shown to be involved in tumor progression by inhibiting the activity of CD4^+^ and CD8^+^ T cells, by the production of arginase and reactive oxygen species (ROS) and by inducing Tregs through an IL-10 and IFN-*γ*-dependent process. Their interaction with macrophages could result in the induction of the type 2 phenotype due to increased IL-10 secretion.

### Peripheral blood markers reflecting the immunologic tumor microenvironment

One possibility to analyze the aggressiveness of OSCC is the identification of prognostic biomarkers in the peripheral blood. Indeed, a number of circulating biomarkers have been described [96]. These include e.g. circulating peripheral blood CD14+/CD16+ monocyte-derived macrophages (MDMs), which have been evaluated in OSCC patients by Grimm and co-workers and characterized as CD14^+^/CD16^+^ MDMs [97]. Moreover, the lowest ratio of IL-17F/VEGF was found in OSCC patients (P  <  0.05): The lower ratio of IL-17F/VEGF correlated to higher tumor stage and lymph node metastases. Furthermore, the serum level of IL-17F and the ratio of IL-17F/VEGF were positively associated with the number of CD3^+^ CD4^+^ T cells. These data indicated that serum IL-17F might originate from PBMCs during the development of OSCC and thus could be used to distinguish OSCC patients from healthy individuals [98]. The proportion of CD57^+^ T cells, including both CD8^+^ and CD4^+^ subsets significantly increased with clinical stage, especially in parallel with tumor size as described by Iida and co-authors. The population of CD57^+^ T cells is another potent prognostic marker and may also influence the systemic immunity of patients with OSCC [99]. Moreover, different serum levels of IL17A, TGFβ1, IL4 and IL10 were significantly higher in oral cancer patients, while the concentration of IL2 and IFN-γ was relatively lower in patients when compared to controls. TGFβ1 levels significantly correlated with disease. In this context, IL17A might represent a risk factor for OSCC [100].

## Characteristics of the immune escape in OSCC

It has been proposed that OSCC could escape the anti-tumor response by several distinct mechanisms. So far alterations in the HLA class I molecules due to deficient expression of components of the antigen processing machinery (APM) has been mainly described thereby leading to resistance to CTL-mediated lysis. In HNSCC a coordinated downregulation of various APM components, in particular TAP1, TAP2, tapasin and HLA class I antigens, were found with higher frequency of downregulation in metastasis when compared to the primary lesions of the same patients [101, 102]. This was further associated with a worse clinical outcome of patients. Furthermore, a downregulation of HLA class I antigens and of most APM components in OSCC lesions was described when compared to adjacent normal tissues. The deficient expression of the low molecular weight protein LMP2, a component of the proteasome, was associated with a reduced CD8^+^ T cell infiltration, which was associated with the presence of regional lymph node metastases and with reduced survival rates of these patients. The molecular mechanism of APM deficiencies in OSCC could be diverse and include ganglioside-mediated or transcriptional-mediated down-regulation of several HLA class I APM components [103, 104]. In addition a lack of IFN-γ inducibility has been shown as well as defects in the IFN-γ signal transduction pathway, such as impaired phosphorylation of one or more components, which also negatively interfere with the constitutive HLA class I APM component expression.

Furthermore, the development of OSCC is strongly influenced by the host immune system, such as reduced frequency of (activated) immune effector cells, increased frequency of regulatory T cells (Treg), functional defects or apoptosis of both circulating and tumor-infiltrating T cells and the local microenvironment disabling TIL due to absent or low expression of the CD3 zeta chain (CD3*ζ*), decreased proliferation in response to mitogens or IL-2, an imbalance in the cytokine profile and pronounced apoptotic features [105–108]. Moreover, immune cell dysfunction is also found in peripheral circulating mononuclear cells of patients with advanced OSCC. HNSCC cells also produce high quantities of TGF-*β*1, which reduces the expression of NK cell receptor NKG2D and CD16 and inhibits the biological functions of NK cells. In addition, an accumulation of Tregs and TAMs in the TILs and/or of peripheral blood mononuclear cells in head and neck cancer patients was detected, which could be related to the early recurrence and patient’s prognosis [109–111]. High levels of various soluble mediators were found in the peripheral blood and/or tumor microenvironment of HNSCC, such as VEGF, PGE2, TGF-*β*, IL-6, and IL-10. These factors have been shown to inhibit the immune response at different levels.

Accumulating evidence exists that myeloid-derived cells including myeloid derived suppressor cells, polymorphonuclear granulocytes (PMN) and dendritic cells play an important role in tumor angiogenesis [112–114]. The frequency and function of these cells are modulated by tumor-derived factors thereby increasing the inflammatory or immune suppressive activity [115]. In addition, OSCC have also a significant impact on dendritic cells (DCs). A larger number of DCs in non-metastatic lymph nodes was found when compared to metastatic lymph nodes of OSCC. The immature DC marker CD1a was especially present in the cancer “nest”, whereas the mature DC marker CD83 was more prominent in the peritumoral area. The relationship between the expression of VEGF and DC infiltration, which plays an important role in immune defense against tumors, remains unclear. However, VEGF-A expression is not only involved in tumor angiogenesis, and disease progression but also in immune suppression by inhibiting the differentiation of CD1a^+^ immature DCs from progenitor cells leading to a reduced number of mature DCs and increased levels of dysfunctional CD83^+^ mature DCs [116]. Moreover, in OSCCs a greater number of S100^+^ and CD1a^+^ immature DCs in adjacent tissue and regional lymph nodes in patients without metastasis had been found. In contrast, CD83^+^ mature DCs were more abundant in patients with metastasis [117].

Tumor cells can modulate the expression of toll like receptors (TLRs) present on the surface of immune cells. Monocyte-derived DCs (MDCs) of OSCC patients express all TLRs except TLR4, -9, and -7 [118–120]. Thus altered TLR expression is an important tumor-promoting event in OSCC progression. OSCCs can also influence the frequency of circulating MDC and plasmacytoid dendritic cell populations. The number of circulating MDCs (LIN-DR^+^CD11c^+^) was significantly lower in patients with OSCC. However, the circulating MDC population increased after removal of the tumor suggesting that this reduction was reversible and controlled by the presence of tumor cells. In addition, TAMs are involved in the angiogenesis and tumor progression of OSCC as described by various groups. The number of TAMs as determined by immunohistochemical analysis using the CD68 antibody is higher in carcinomas [121]. Comparison of TAMs with clinical parameters demonstrated an association between their frequency, tumor stage, grade and invasion, intratumoral microvessel density and the presence of angiogenic factors, such as VEGF [122]. This was further confirmed by the analysis of the expression of cell cycle (cyclin E and p53) and proliferation markers (Ki-67) as well as macrophage infiltration [123]. A direct correlation between the macrophage infiltration and the tumor proliferation index was noted, which suggested that the number of TAMs is functionally linked to tumor progression [124]. In addition macrophages play a role in OSCC formation by contributing to neovascularization. In fact, OSCCs could attract macrophages by secreting MCP-1 and TGF-*β*1 [125, 126]. The mechanisms responsible for T cell apoptosis in OSCC patients involve the Fas/FasL or the TRAIL and TNF-α signaling pathways. The FasL expression was found on the cell surface of OSCC cells leading to an apoptotic signal of circulating Fas^+^ T lymphocytes. The suppression of Treg might depend on Fas/FasL-mediated apoptosis: CD4^+^ T cells were resistant to Fas-mediated apoptosis by Tregs, but were able to induce Treg apoptosis in the presence of low concentrations of IL-2. The expression of monocyte chemotactic protein-1 (MCP-1/CCL2) and macrophage inflammatory protein-1α (MIP-1α/CCL3) was found in OSCC lesions and might also control disease progression. In addition, serum levels of CCL2 and CCL3 in OSCC were determined. A significant lower concentration of CCL2 was detected in the OSCC patients when compared to that in the healthy controls. Serum levels of CCL3 were positively related to the tumor size, while the CCL2/CCL3 ratio in OSCC patients was correlated to TNM (tumor, node, metastasis) [127, 128]. Thus CCL2 and CCL3 are associated with progression of OSCC and might serve as potential biomarkers.

## Clinical relevance of the frequency and composition of immune cell infiltrates

Recently, the prognostic value of various tumor-infiltrating immune cells populations was determined in OSCC patients. Interestingly, a high frequency of CD4^+^CD69^+^ T cells was linked to a better prognosis, and CD4^+^Foxp3^+^ T cells were positively correlated with better locoregional control. Moreover, a higher density of CD4^+^CD25^+^ Tregs was also linked to a good prognosis in OSCC. In discrepancy to these studies the presence of Tregs in TILs was linked to a worse prognosis in OSCC patients in other reports. Suppression by the tumor microenvironment is mediated by a unique subset of CD4^+^CD25^high^Foxp3^+^ Tregs that produce IL-10 and TGF-*β*, which lead to a more antiproliferative effects [108, 129, 130]. Changes in the expression of the *ζ* chain of TILs are biologically significant because the absence or low expression of this chain in TILs in patients with stage III or IV HNSCC predicts a poor survival compared with patients expressing a normal *ζ* chain. The importance of the *ζ* chain was further confirmed by demonstrating a lower expression of the *ζ* chain in circulating CD4^+^ and CD8^+^ T cells and CD3^−^CD56^+^CD16^+^ NK cells in the blood of patients with OSCC when compared to healthy individuals. Reichert and co-authors studied the DC population and the expression of the *ζ* chain in TILs in a large series of 132 OSCCs [131]. A low density of DCs and absent or low expression of the *ζ* chain in TILs was correlated with a poor prognosis of survival and a high risk of recurrence. The more advanced cases demonstrated higher rates of Tregs and B cells and fewer CD8^+^ T cells. In the low-risk group, a high concentration of CD20^+^ TILs was linked to a better survival rate, whereas this increase was linked to a worse prognosis in the high-risk group.

## Conclusions

Although hypoxia has been associated with an increased immune escape a deeper understanding of the factors that cause immune suppression in OSCCs might be relevant for the development of novel anti-cancer therapies. The worse prognosis of these patients has been linked to hypoxia and hypoxia-induced immune escape. Impaired anti-tumor responses of OSCC patients are caused by the tumor itself, by the presence of functional defects or apoptosis of both circulating and tumor-infiltrating T cells, but also by soluble factors of the tumor microenvironment including soluble factors and the hypoxic microenvironment, which leads to an accumulation of immune suppressive cells, like TAM, Tregs and MDSCs macrophages as well as a downregulation in the function and activity of T lymphocytes and DCs.

## Abbreviations

ANG: angiogenin
APM: antigen-processing machinery
ARNT: aryl hydrocarbon receptor nuclear translocator
CA: carbonic anhydrase
CEA: carcinoembryonal antigen
COX: cyclooxygenase
CTL: cytotoxic T lymphocytes
EGF: epidermal growth factor
EMT: epithelial in mesenchymal transition
FIH-1: factor inhibiting HIF-1 alpha
GM: granulocyte macrophage
HIF: hypoxia-inducible factor
HRE: hypoxia responsive element
IL: interleukin
LYVE-1: lymphatic vessel endothelial hyaluronan receptor 1
MCT-1: monocarboxylate transporter
MDSC: myeloid-derived suppressor cell
MHC: major histocompatibility complex
MVD: microvessel density
NHE1: solute carrier family 9 member A1
OSCC: oral squamous cell carcinoma
OSR: overall survival rate
PDH: prolyl hydroxylase
PDL-1: programmed death-ligand 1
PG: prostaglandin
pHe: extracellular pH
pHi: intracellular pH
PTG: prostaglandin-endoperoxide
SCC: squamous cell carcinoma
TAMs: tumor-associated macrophages
TGF: transforming growth factor
TILs: tumor-infiltrating lymphocytes
TNF: tumor necrosis factor
TNM: tumor-node metastasis
Tregs: regulatory T cells
pVHL: von Hippel-Lindau tumor suppressor protein
VEGF: vascular endothelial growth factor
