# Correction to: Clinical relevance of the tumor microenvironment and immune escape of oral squamous cell carcinoma

**DOI:** 10.1186/s12967-018-1407-9

**Published:** 2018-02-28

**Authors:** Alexander W. Eckert, Claudia Wickenhauser, Paul C. Salins, Matthias Kappler, Juergen Bukur, Barbara Seliger

**Affiliations:** 1 Department of Oral and Maxillofacial Plastic Surgery, Martin-Luther-University Halle-Wittenberg, Ernst-Grube-Str. 40, 06120 Halle (Saale), Germany; 2 Institute of Pathology, Martin-Luther-University Halle-Wittenberg, Magdeburger Str. 8, 06110 Halle (Saale), Germany; 3 Mazumdar Shaw Cancer Center and Narayana Hrudayalaya Multi Specialty Hospital, 258/A, Bommasandra Industrial Area, Bangalore, 560099 India; 4 Institute of Medical Immunology, Martin-Luther-University Halle-Wittenberg, Magdeburger Str. 2, 06110 Halle (Saale), Germany

## Correction to: J Transl Med (2016) 14:85 10.1186/s12967-016-0828-6

The original version of this article [1], published on 5 April 2016, contains a mistake. In the ‘Role of pH stabilisation’ section, “intracellular pH” has been incorrectly abbreviated as “pHe”. The correct abbreviation is “pHi”. The affected sentence with the correct abbreviation is given below.

- The metabolic adaption accumulates different ionic exchangers at the tumor cell membrane to maintain intracellular pH (pHi) (Fig. 2).

The figure which is cited in the above sentence can be reviewed in the original article.
